# Changing trends in the incidence (1999-2011) and mortality (1983-2013) of cervical cancer in the Republic of Korea

**DOI:** 10.4178/epih/e2015024

**Published:** 2015-05-29

**Authors:** Yoon Park, Champadeng Vongdala, Jeongseon Kim, Moran Ki

**Affiliations:** Department of Cancer Control and Policy, Graduate School of Cancer Science and Policy, National Cancer Center, Goyang, Korea

**Keywords:** Uterine cervical neoplasms, Cervix uteri, Incidence, Mortality, Age groups, Korea

## Abstract

Cervical cancer is a well-known preventable cancer worldwide. Many countries including Korea have pursued the positive endpoint of a reduction in mortality from cervical cancer. Our aim is to examine changing trends in cervical cancer incidence and mortality after the implementation of a national preventive effort in Korea. Cervical cancer incidence data from 1999 to 2011 and mortality data from 1983 to 2013 were collected from the Korean Statistical Information Service. Yearly age-standardized rates (ASR) per 100,000 were compared using two standards: the 2005 Korean population and the world standard population, based on Segi’s world standard for incidence and the World Health Organization for mortality. In Korea, the age-standardized incidence of cervical cancer per 100,000 persons declined from 17.2 in 2000 to 11.8 in 2011. However, the group aged 25 to 29 showed a higher rate in 2011 (ASR, 6.5) than in 2000 (ASR, 3.6). The age-standardized mortality rate per 100,000 persons dropped from 2.81 in 2000 to 1.95 in 2013. In the worldwide comparison, the incidence rates remained close to the average incidence estimate of more developed regions (ASR, 9.9). The decreasing mortality trend in Korea approached the lower rate observed in Australia (ASR, 1.4) in 2010. Although the incidence rate of cervical cancer is continuously declining in Korea, it is still high relative to other countries. Moreover, incidence and mortality rates in females aged 30 years or under have recently increased. It is necessary to develop effective policy to reduce both incidence and mortality, particularly in younger age groups.

## INTRODUCTION

Globally, cervical cancer is a preventable cancer [1]. It is causally associated with infection with the oncogenic human papillomavirus (HPV), particularly the two most common strains, HPV 16 and 18 [2-4]. The development of various preventive strategies and clinical approaches has achieved a reduced incidence of cervical cancer, and hence mortality, in a number of developed countries [5]. Previous studies have shown that the introduction of a screening program plays a critical role in the control of cervical cancer [6].

In 1988, Korea introduced the practice of population-based cervical cancer screening using the Papanicolaou (Pap) smear, initially providing this service to selected people insured by the Korea National Health Insurance Program [4]. The National Cancer Screening Program was established in 1999 and has since provided free biennial Pap smear tests for females aged over 30 years. In 2000, the National Health Insurance Law mandated the Pap test as part of a nationwide screening program [7-9]. Several studies have reported positive outcomes related to cervical cancer screening that show a significantly lower incidence of invasive cervical cancer (International Classification of Diseases, 10th revision [ICD-10]: code C53) and carcinoma *in situ* (CIS; ICD-10: D06) among Korean females who were screened two or more times than among unscreened females [10]. In short, since around 2000, the implementation of a population-based screening program has reduced the overall cervical cancer incidence and mortality rates in Korea. However, there may be different trends in age groups that could influence the incidence and mortality rates.

The aim of the present study was to examine changing trends in cervical cancer incidence and mortality in Korea. Here, we discuss patterns in both incidence and mortality with respect to yearly comparisons in specified age groups and to a worldwide comparison.

## MATERIALS AND METHODS

Cervical cancer was classified according to the International Classification of Diseases for Oncology, 3rd edition [11], and converted according to the ICD-10 [12]. The code for invasive cervical cancer is C53, classified as malignant neoplasm of cervix uteri. Statistical data in this study only included cases of invasive cervical cancer. Incidence and mortality data were collected directly from the Korean Statistical Information Service [13] and indirectly from the Korea Ministry of Health and Welfare, the National Health Insurance Service, and the Korean Central Cancer Registry [14,15]. Incidence and mortality rates were compared by age from 1999 to 2011 and from 1983 to 2013 respectively. Both incidence and mortality data were gathered from the online database that showed selected datasets after choosing the type of cancer, required statistical unit, age, sex, and duration of time. Age-standardized rates calculated using the 2005 Korean population (ASR, K), were mainly used to compare differences in yearly rates. The reference population data were collected from the Korean Statistical Information Service [16]. To analyze the age-specific incidence trends from 2000 to 2010, ASRs from three different years (2000, 2005, and 2010) were used.

For the worldwide comparison, age-standardized incidence rates in developed regions as classified by the GLOBOCAN project were collected as follows: all regions of Europe, Northern America, Australia, New Zealand, and Japan [17]. We selectively collected data from the list of countries in the databases of Cancer Incidence in Five Continents (CI-5) [18]: France (eight registries), United Kingdom (nine registries), United States (Surveillance, Epidemiology, and End Results, nine registries), Canada (three registries), Australia (six registries), New Zealand and Japan (three registries). Since the available data were limited until 2007, the estimated incidence data from the 2012 GLOBOCAN project were also collected [17]. Incidence rates in Korea (Table 1) were converted based on Segi’s world standard population (ASR, W) [19,20]. When comparing global trends in mortality rates, age-specific data were only available from three of the developed countries mentioned above. We collected data directly from the New Zealand Cancer Registry [21], the Australasian Association of Cancer Registries [22], and the National Cancer Center in Japan [23]. For mortality, ASRs were determined using the World Health Organization (WHO) world standard population (ASR, W) [24].

## RESULTS

### Incidence rates of cervical cancer

**Figure 1.**
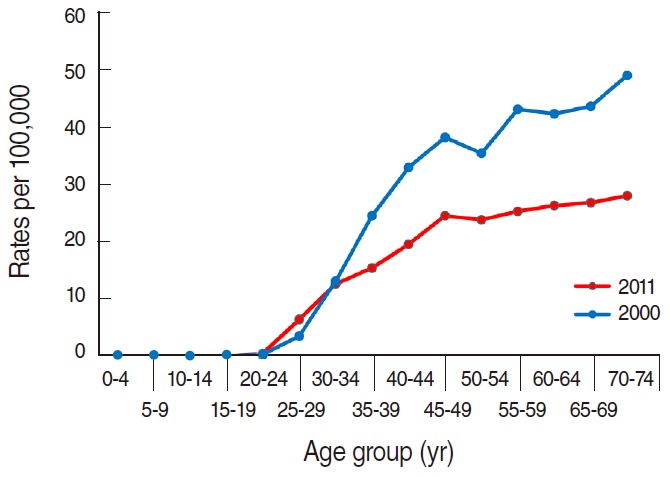
Age-specific incidence rates per 100,000 populations of cervical cancer in Korea, 2000 and 2011. Source from Korean Statistical Information Service. Number of cancer patients, relative frequency, crude rate, age-adjusted incidence by cancer site and sex from 1999 to 2011; 2014 [25].

In Korea, the incidence rates of cervical cancer have significantly declined since 2001. Specifically, the incidence has declined from 4,443 cases in 1999 to 3,760 cases in 2011. Crude rates and ASRs per 100,000 decreased from 18.9 and 18.6, respectively, in 1999 to 15.0 and 11.8 in 2011 (Table 1). Compared to the year 2000, a lower incidence was shown in all age groups in 2011 except in those aged under 30. Individuals aged 25 to 29 showed a higher incidence of 6.5 in 2011 compared to 3.6 in 2000 (Figure 1) [25].

**Figure 2.**
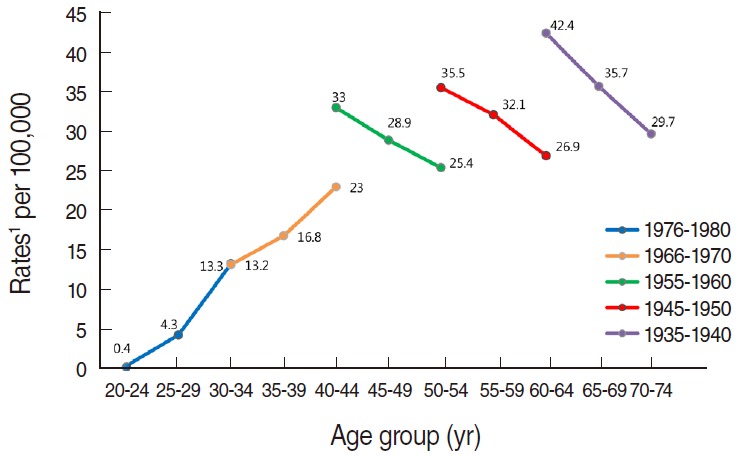
Trends in age-specific incidence rates of cervical cancer by birth cohort group in Korea, 2000, 2005 and 2010. Source from Korean Statistical Information Service. Number of cancer patients, relative frequency, crude rate, age-adjusted incidence by cancer site and sex from 1999 to 2011; 2014 [25]. ^1^Age-adjusted to the 2005 Korea standard population.

In Figure 2, each birth cohort is represented at three points, depicting the incidence in 2000, 2005, and 2010. The birth cohort of 1976 to 1980 and the cohort of 1966 to 1970 showed similar incidences at ages 30 to 34 of 13.3 and 13.2 respectively. Older age groups showed higher incidences if they came from earlier birth cohort groups. Specifically, between the ages of 40 and 44, there was a higher incidence of 33.0 in the 1955 to 1960 birth cohort compared to that of 23.0 in the 1966 to 1970 cohort. Likewise, the rate of 35.5 in the 1945 to 1950 cohort was higher than that of 25.4 in the 1955 to 1960 cohort for individuals aged 50 to 54, and the rate of 42.4 in the 1935 to 1940 cohort was higher than that of 26.9 in the 1945 to 1950 cohort for individuals aged 60 to 64.

**Figure 3.**
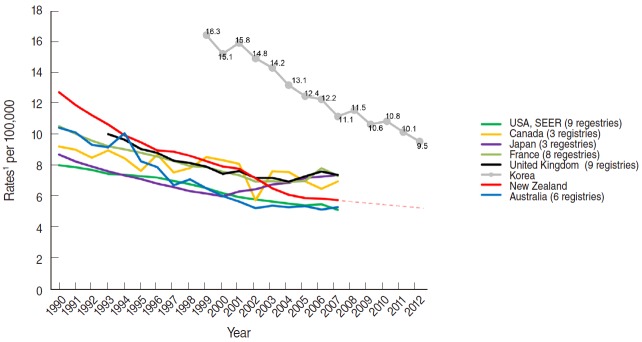
Trends of incidence rates of cervical cancer, worldwide, 1990-2012^2^. Source from International Agency for Research on Cancer. GLOBOCAN 2012: estimated cancer incidence, mortality and prevalence worldwide in 2012; 2014 [17]; International Agency for Research on Cancer. Cancer incidence in five continents volume X(CI5X); 2013; 2014 [18]. SEER, Surveillance, Epidemiology, and End Results. ^1^Age-adjusted to Segi ‘s world standard population; ASR, age-standardized rates. ^2^Estimated incidence rate in ASR (W), 2012: the estimates incidence rates in the year of 2012 are representable for each country as a whole while the data from CI-5 were collected selectively by cancer registry regions.

For the worldwide comparison, converted rates using Segi’s world population appear lower than the rates using the 2005 Korean population, such that incidence changes from 18.6 per 100,000 (ASR, K) to 16.3 per 100,000 (ASR, W) in 1999 and from 11.7 per 100,000 (ASR, K) to 10.1 per 100,000 (ASR, W) in 2011. Compared to more developed countries, incidence rates in Korea are relatively high (Figure 3). For instance, the ASR of 12.4 per 100,000 in 2005 is similar to that of New Zealand in 1990 (ASR, W: 12.65 per 100,000), which was the highest reported rate in 1990 among comparable countries. From the 1990s to 2007, all other countries compared showed lower incidence than Korea, and showed a steadily decreasing pattern during this time. The GLOBOCAN project estimated the cervical cancer incidence rates in 2012. Due to the different regional classifications used in the GLOBOCAN and CI-5, only two among eight countries could be compared in this study (Figure 3): an ASR of 9.5 per 100,000 in Korea and 5.3 per 100,000 in New Zealand.

### Mortality rates of cervical cancer

In Korea, the annual mortality increased from 129 in 1983 to 892 in 2013. Crude rates and ASRs per 100,000 changed from 0.7 and 0.98, respectively, in 1983 to 3.5 and 1.95 in 2013 (Table 1). However, when interpreting these mortality data, it should be understood that a limitation of this study is the use of uncorrected mortality records from death certificates that misclassified cervical cancer in Korea. A study conducted in 2007 corrected the number of cervical cancer deaths by comparing death certificates from cases containing data on unspecified uterine cancer with the national cancer incidence database of the entire cancer registry in Korea. After the correction, there was an apparent reduction in mortality; the overall age-standardized mortality rate per 100,000 persons decreased from 5.2 in 1993 to 3.9 in 2002, based on the 1967 WHO world standard population [4]. From 2003 to 2013, there was a gradual reduction of cervical cancer mortality in Korean females.

**Figure 4.**
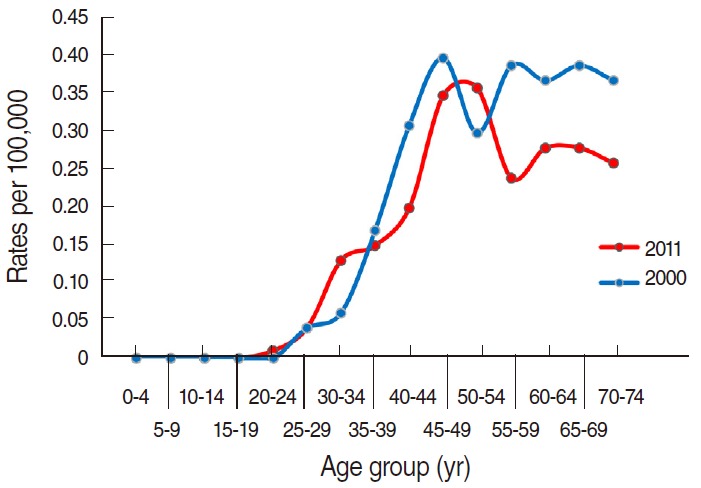
Age-specific mortality rates per 100,000 populations of cervical cancer in Korea, 2000 and 2011. Source from Korean Statistical Information Service. Death causes by 5-year age group and gender, mortality rates (1983-2013); 2014 [13].

Groups aged 50 and over generally had a higher proportion of overall mortality from cervical cancer. From the ages of the mid-30s to mid-40s, the mortality rates increased significantly. During the years from 2000 to 2011, mortality rates were not changed or improved in any age group except in the older groups aged in the mid-50s and above. In certain age groups, mortality rates were higher in the more recent year of 2011 than in 2000. The age groups of 30 to 34 and 50 to 54 showed significantly higher mortality rates in 2011, with 0.13 per 100,000 and 0.36 per 100,000, respectively, compared to 0.06 per 100,000 and 0.30 per 100,000 in 2000 (Figure 4).

**Figure 5.**
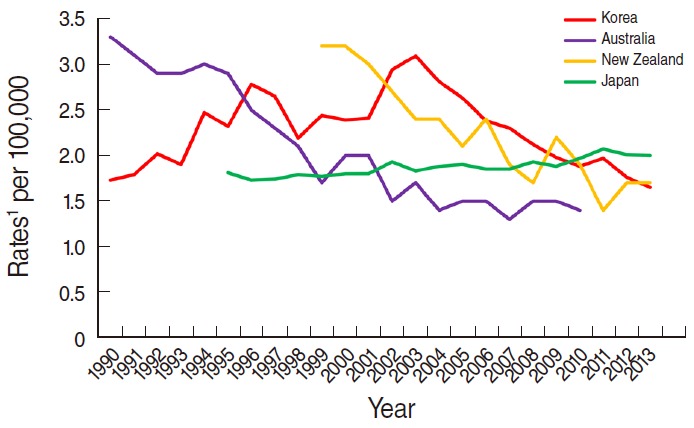
Trends of mortality rates of cervical cancer, worldwide, 1990-2013. Source from Korean Statistical Information Service. Death causes by 5-year age group and gender, mortality rates 1983-2013; 2014 [13]; Ministry of Health. Cancer: historical summary 1948-2011; 2014 [21]; Australian Institute of Health and Welfare. Australian cancer incidence and mortality (ACIM) book for cervical cancer; 2014 [22]; Japan National Cancer Center. Cancer mortality from vital statistic in Japan 1958-2013; 2014 [23]. ^1^Age-adjusted to World Health Organization world standard population.

For the worldwide comparison, three countries including Korea showed a decreasing pattern over the years studied (Figure 5). For instance, the ASR in Australia of 3.1 per 100,000 in 1991 was similar to that of Korea in 2003 and of New Zealand in 2001 (ASR, W: 3.09 per 100,000 and 3.0 per 100,000, respectively); these different years reflect a trend in each country of decreasing mortality rates. Notably, although the mortality rates showed a gradual decline in Korea, the mortality rate in 2013 (ASR, W: 1.65 per 100,000) was still higher than that of Australia in 2010 (ASR, W: 1.4 per 100,000).

Since 2010, a pattern of slightly increasing mortality has been observed in Japan, which first reported a higher mortality rate (ASR, W: 1.97 per 100,000) than that of Korea (ASR, W: 1.88 per 100,000) in 2010.

## DISCUSSION

Both incidence and mortality rates of cervical cancer in Korea have been declining since the nationwide cancer screening program was implemented [10]. Despite a decrease in cervical cancer cases, the age-standardized incidence standardized against the Korean population is higher than the age-standardized incidence using the world standard population. This is due to the relatively higher distribution of the elderly in Korea compared with the demographics of the world population.

In Korea, invasive cervical cancer appears to have increased predominantly among the younger age group [26]. The increasing incidence in individuals aged 20 to 29 and relatively slight reduction in mortality in all age groups might be the result of cases with more advanced cancer at diagnosis and consequent poor prognosis and low survival rates [27,28]. Since cure rates depend strongly on the stage at diagnosis [29], detection at an advanced stage might be closely related to unchanged mortality rates over time. At this late stage, cancer detection might occur at voluntary hospital visits after observation of relevant signs or symptoms [30] rather than by regular screening tests.

The significantly high mortality in groups aged in the late 30s, late 40s, and above may reflect potential risk factors. Early initiation of sexual activity might cause early exposure to HPV infection [31]. Females infected with an oncogenic HPV type will progress to high-grade cervical intraepithelial neoplasia in their 20s or early 30s. The latency from HPV infection to cancer development is long, such that invasive cancer may appear 10 or more years after infection [29]. Moreover, elderly females might receive more exposure to low hygiene environments that increase the risk of HPV infection [32] and have less opportunity to engage with preventive programs from a young age. Despite the introduction of HPV vaccination, the current vaccines do not cover all potential oncogenic HPV types [29] and vaccination does not prevent cancer among individuals already infected with HPV.

In countries where organized cytological screening has been implemented over the last three or four decades, cervical cancer incidence and mortality rates have declined steadily [33, 34]. In the US, for example, incidence has been falling at a consistent rate across most states, declining by 1.9% per year. Overall mortality from cervical cancer has also been falling at a consistent rate, and this pattern in mortality is similar to that of Korea at present [35]. To obtain a reduction in mortality, the US has provided free or low-cost cervical cancer screening and health services for more than 20 years. Moreover, it has made a continuous effort to increase the proportion of cervical cancer screening from 83% of the female population in 2010 to the Healthy People 2020 target of 93% [36]. It has also emphasized that nonfinancial barriers, such as lack of awareness of disease, need to be addressed to save more lives [37].

Exploring changing trends in cervical cancer raises an important question about the effectiveness of cervical cancer screening. Routine screening might not be an efficient sole means of protecting against cervical cancer, especially in the younger age group of 20 to 40 years in Korea. A combination of public awareness of causal factors, early cancer detection by regular screening with the incorporation of HPV DNA testing, and vaccination at reasonable costs will constitute key preventive tools against cervical cancer [38] in Korea, as well as on a global scale.

## Figures and Tables

**Table 1.** Incidence rate (1999-2011) and mortality rate (1983-2013) by year of cervical cancer in Korea

Year	Incidence			Mortality		
	Cases	Crude rate (per 100.000)	ASR^1^ (per 100.000)	Cases	Crude rate (per 100.000)	ASR^1^ (per 100.000)
1983	-	-	-	129	0.7	0.98
1984	-	-	-	147	0.7	1.09
1985	-	-	-	177	0.9	1.26
1986	-	-	-	189	0.9	1.26
1987	-	-	-	222	1.1	1.49
1988	-	-	-	265	1.3	1.67
1989	-	-	-	359	1.7	2.21
1990	-	-	-	343	1.6	2.07
1991	-	-	-	360	1.7	2.11
1992	-	-	-	439	2.0	2.38
1993	-	-	-	425	1.9	2.25^2^
1994	-	-	-	560	2.5	2.91^2^
1995	-	-	-	554	2.4	2.76^2^
1996	-	-	-	668	2.9	3.28^2^
1997	-	-	-	680	2.9	3.11^2^
1998	-	-	-	610	2.6	2.56^2^
1999	4,443	18.9	18.6	690	2.9	2.87^2^
2000	4,253	18.0	17.2	726	3.1	2.81^2^
2001	4,572	19.2	18.0	807	3.4	2.84^2^
2002	4,402	18.4	16.8	1,009	4.2	3.42^2^
2003	4,373	18.2	16.2	1,111	4.6	3.60
2004	4,130	17.1	14.9	1,078	4.5	3.28
2005	4,014	16.5	14.1	1,066	4.4	3.05
2006	4,047	16.6	13.9	1,002	4.1	2.78
2007	3,755	15.3	12.7	987	4.0	2.72
2008	4,004	16.2	13.2	954	3.9	2.48
2009	3,803	15.3	12.2	950	3.8	2.32
2010	3,956	15.9	12.6	956	3.8	2.21
2011	3,760	15.0	11.8	989	4.0	2.31
2012	-	-	-	889	3.5	2.06
2013	-	-	-	892	3.5	1.95

Source from Korean Statistical Information Service. Death causes by 5-year age group and gender, mortality rates (1983-2013); 2014 [13]; Korean Statistical Information Service. Number of cancer patients, relative frequency, crude rate, age-adjusted incidence by cancer site and sex from 1999 to 2011; 2014 [25]. ASR, age-standardized rates.

1 ASR per 100,000 using the Korea population of 2005.

2 Corrected mortality using national death certification data. ASR using direct method based on 1967 World Health Organization world standard population (from 1993 to 2002 by year: 5.20, 5.60, 5.10, 5.10, 4.90, 4.30, 4.20, 4.10, 4.00, 3.90, respectively). Adapted from Shin HR, et al. Int J Cancer 2008;122:393-397 [4].
